# Scan speed affects quantitative optical coherence tomography angiography vascular metrics

**DOI:** 10.1038/s41598-024-80562-4

**Published:** 2024-11-22

**Authors:** Lourdes Vidal-Oliver, Elisa Herzig-de Almeida, Sabrina Spissinger, Rosa Dolz-Marco, Robert P. Finger

**Affiliations:** 1 Department of Ophthalmology, University Hospital Mannheim & Medical Faculty of Mannheim, University of Heidelberg, Theodor-Kutzer-Ufer 1-3, 68167 Mannheim, Germany; 2 Unit of Macula, Oftalvist Clinic, C/ Ruzafa 19, 46004 Valencia, Spain

**Keywords:** Optical coherence tomography angiography, Scan speed, A-scan rate, Vessel density, Vessel length density, Diagnostic markers, Image processing

## Abstract

Optical coherence tomography angiography (OCTA) offers the possibility of obtaining objective quantification of retinal vasculature, with increasing utility as biomarkers for both systemic and ocular diseases. However, the differences between different manufacturers and scan settings are still an important limitation, as many parameters could affect vessel quantification. Here we aim to study the influence of scan speed on quantitative vascular parameters using OCTA. 57 eyes in good retinal health received two consecutive 20 × 20° macular OCTA scans using SPECTRALIS SHIFT at different scan speeds: 85 and 125 kHz. Vessel density (VD) and vessel length density (VLD) in both the superficial (SVP) and deep vascular plexus (DVP), duration of the scan and Q-value were compared between scanning speeds. OCTA images taken at 85 kHz showed significantly higher VD and VLD values (% reduction in SVP: VD -4.03% to -5.8%, VLD − 4.96% to -6.07%; in DVP: VD -3.35% to -6.58%, VLD − 3.60% to -6.66%). At 125 kHz, acquisition time was reduced by 22%, but Q-values were lower (34.1 vs. 35.6). Thus, while higher A-scan rates reduce acquisition time, they lower VD and VLD in both plexus. Further studies in eyes with pathology are needed to better understand the magnitude of these changes.

## Introduction

Optical coherence tomography angiography (OCTA) is a useful extension of OCT that has been introduced into clinical practice in recent years. OCTA enables three-dimensional reconstruction of vessel structure by detecting blood flow as decorrelation changes between different scans of the same area of the retina at different time points.^1^ Compared to fluorescein angiography, OCTA allows a non-invasive assessment of the retinal vasculature up to the midperiphery with the majority of commercially available devices.^2^ However, patient cooperation is required to obtain high-quality images that can be properly interpreted. Since its introduction, technical improvements have been made to overcome some of the pitfalls, such as the field of view. To image a large retinal area with commercially available OCTA devices, longer scan times are required, resulting in image artifacts due to eye movements. Faster scanning speeds (with shorter interscan time) can reduce the likelihood of eye movement artifacts, but at the cost of reduced sensitivity for slow flow detection.^3^

Changes in various OCTA settings such as image threshold, resolution, sampling rate, interscan time or post-processing algorithms have been shown to affect OCTA interpretation and make comparison between different OCTA manufacturers difficult.^4,5^ The option of the clinician to change scan speed was not widely available but is now being commercialized in the SPECTRALIS OCT platform (Heidelberg Engineering, Heidelberg, Germany). In inter-rater subjective quality assessments, OCTA images acquired at 125 kHz and 85 kHz scan speed were comparable.^6^ However, the effect of scan speed on quantitative vascularization metrics in OCTA remains unclear. As several quantitative OCTA biomarkers are currently being investigated as diagnostic or prognostic factors in various retinal and systemic diseases, how scan speed influences these measurements can be clinically meaningful.^7,8^

In this study, we investigated the effect of two OCTA acquisition speed settings (85 kHz and 125 kHz) on quantitative vascular measurements.

## Methods

In this single-center cohort study, we recruited adult patients with no retinal pathology between March 2024 and May 2024. The study was conducted in accordance with the Declaration of Helsinki and informed consent was obtained from the subjects after explanation of the nature and possible consequences of the study. All experiments were performed in accordance with relevant guidelines and regulations. Ethical approval was obtained from the ethics committee of the University of Heidelberg (Ethik-Kommission II der Universität Heidelberg, Medizinische Fakultät Mannheim, ID number: 2023-666).

We included patients with clear media and good foveal fixation. Exclusion criteria were the presence of astigmatism of > 2 diopters (D) and a spherical error greater than ±4D. We also excluded eyes with other ophthalmological conditions including advanced glaucoma, age-related macular degeneration, vitreoretinal interface abnormalities, inflammatory or retinal vascular diseases.

### Image acquisition protocol

Two consecutive, eye-tracked, 20 × 20° macular OCTA scans (512 A-scans x 512 B-scans) were acquired at different scan speeds (85 and 125 kHz), in random order to avoid fatigue bias. All image acquisitions were made by an ophthalmologist trained in retinal imaging. The duration of each acquisition was recorded with a digital timer. We only included good quality images, defined as imaged with a signal-to-noise ratio (Q-value) higher than 30dB without significant image artifacts (defocus, attenuation or striping). All images were acquired with a SPECTRALIS HRA-OCT3 and the scan speed was changed using the SHIFT technology.

### Image processing and analysis

The *en face* OCTA images of the superficial and deep vascular plexus were imported into Fiji^®^ (ImageJ^®^, Bethesda, USA, v2.14.0/1.54f) for image processing and quantitative analysis. Images were first preprocessed using the Background Subtraction tool, and then binarized using the local Phansalkar auto threshold method.^9,10^ We analyzed vessel density (VD) (calculated as white pixels/image area, expressed in percentage) in 4 areas of 2 mm^2^ each, around the foveal avascular zone (FAZ): nasal, superior, temporal and inferior.^11^ Subsequently, we skeletonized the images and calculated the vessel length density (VLD)(calculated as vessel length per unit area), in the same 4 areas around the FAZ.

To examine the impact of image contrast in OCTA quantification, we performed a subanalysis of the images captured at 125 kHz. Prior to image binarization, image contrast was enhanced using the “Brightness and Contrast” tool in ImageJ^®^ with two different pixel intensity ranges. The lower and upper limits of the intensity ranges were set to 5 and 249, and 10 and 244, respectively. VD and VLD calculations were conducted in the same retinal regions and compared with the baseline image, which was not adjusted for contrast. The aim of this sub analysis was to ascertain the extent of change resulting from contrast alteration and to determine whether differences in VD and VLD caused by scan speed could be mitigated by enhancing contrast.

### Outcome measures

The primary outcome measure was the difference of vascular quantification in *en face* OCTA scans at different scan speeds. For this, we analyzed two parameters: vessel density (VD, %), obtained from binarized images; and vessel length density (VLD, mm^− 1^) obtained from images after skeletonization.

Secondary outcomes included the signal-to-noise ratio (Q-value, dB) and duration of the scan (seconds).

### Statistical analysis

Pairwise comparison of VD, VLD, Q-value and acquisition time at different scan speeds and at different contrast settings were performed using Wilcoxon test. Comparison among the different sectors studied was made using Kruskal-Wallis test. Correlation between Q-value and vessel density values were assessed using Spearman’s r. The statistical significance was set at *p* < 0.05. Multiple comparison correction was performed using the Benjamini-Hochberg method.

## Results

We obtained 61 image pairs at high (125 kHz) and low (85 kHz) scan speed of which we discarded 4 image pairs due to significant attenuation artifacts seen in the en-face images. The final dataset included 57 eyes of 30 patients (14 female, 46.7%) with a mean age of 61.5 years (range 34–81) and a mean spherical error of + 0.4 diopters (D) (range − 3.75 - +3.50 D). Acquisition time from start to end of the exam at 85 kHz scan speed was significantly longer than at 125 kHz (mean 60.7 ± 32.4 vs. 46.4 ± 27.6 s, *p* < 0.0001; % reduction of 22 ± 20.4%). Images taken at 85 kHz had a better signal-to-noise ratio (mean Q-value 85 kHz 35.6 ± 2.3dB vs. 34.1 ± 2.2dB, *p* < 0.0001).

In the overall images, signal-to-noise ratio showed significant positive correlation with VD in the superficial (*r* = 0.38, *p* < 0.0001) and deep vascular plexus (*r* = 0.45, *p* < 0.0001); and with VLD in the superficial (*r* = 0.37, *p* < 0.0001) and deep (*r* = 0.44, *p* < 0.0001) vascular plexus.

**Fig. 1:**
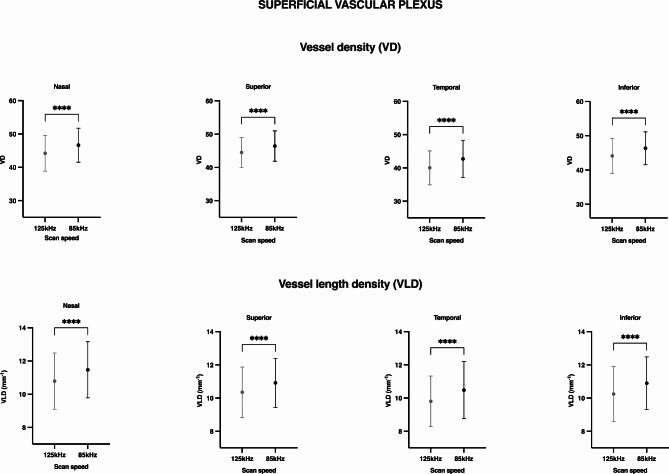
High acquisition speed alters superficial vascular plexus metrics. Mean and standard deviation bars showing vessel density (VD, %) and vessel length density (VLD, mm^-1^) values of the superficial vascular plexus in images taken at 125 kHz and 85 kHz scan speed, in different sectors of the retina. *P-values < 0.05, obtained by Wilcoxon matched-pairs signed rank test and corrected for multiple comparisons.

**Fig. 2:**
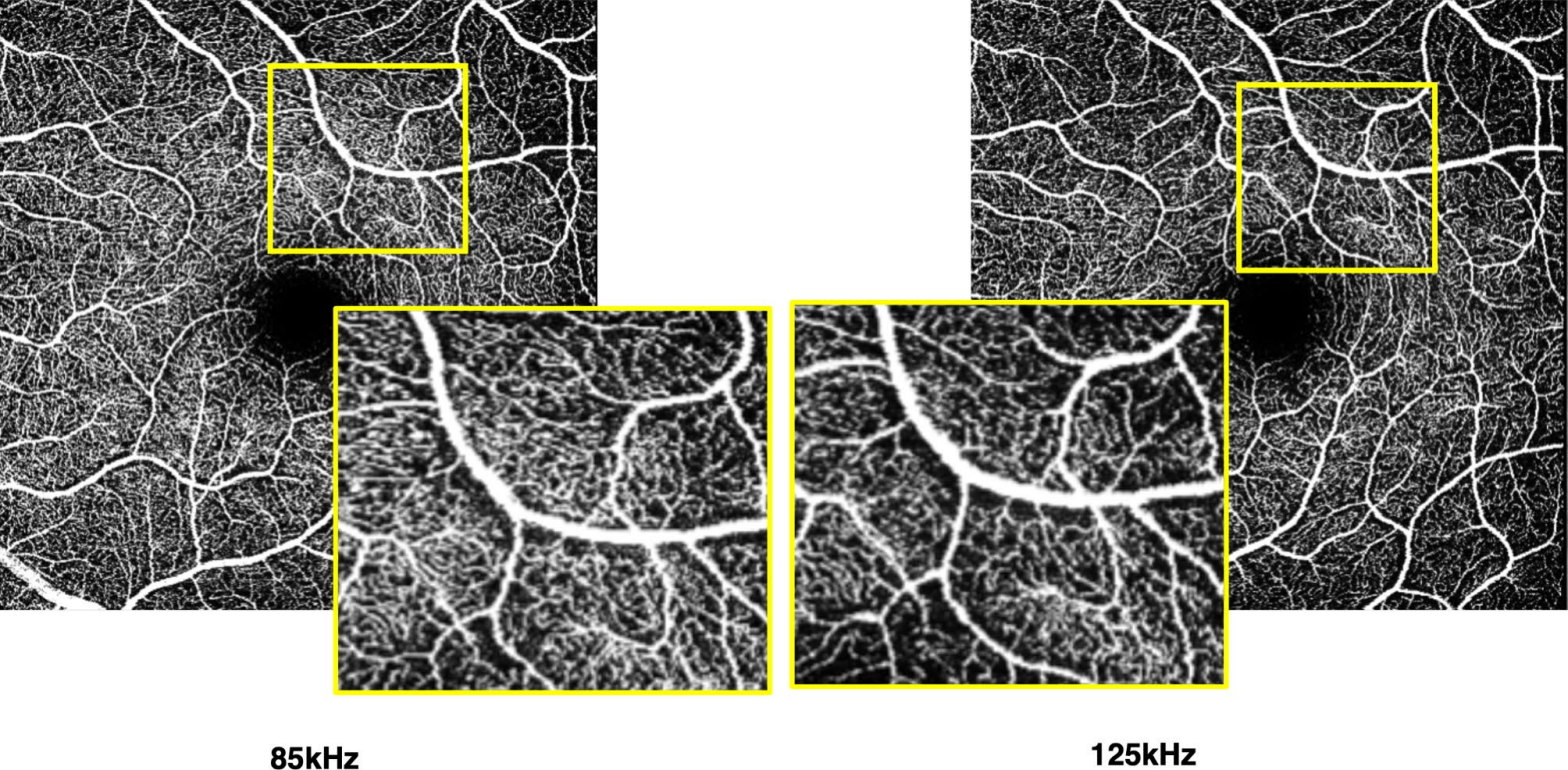
Example of reduced capillary density in rapid acquisition image. *En face* OCTA images of the superficial vascular plexus at 85 and 125 kHz scan speed. Higher density of capillaries can be seen in the left image.

**Fig. 3:**
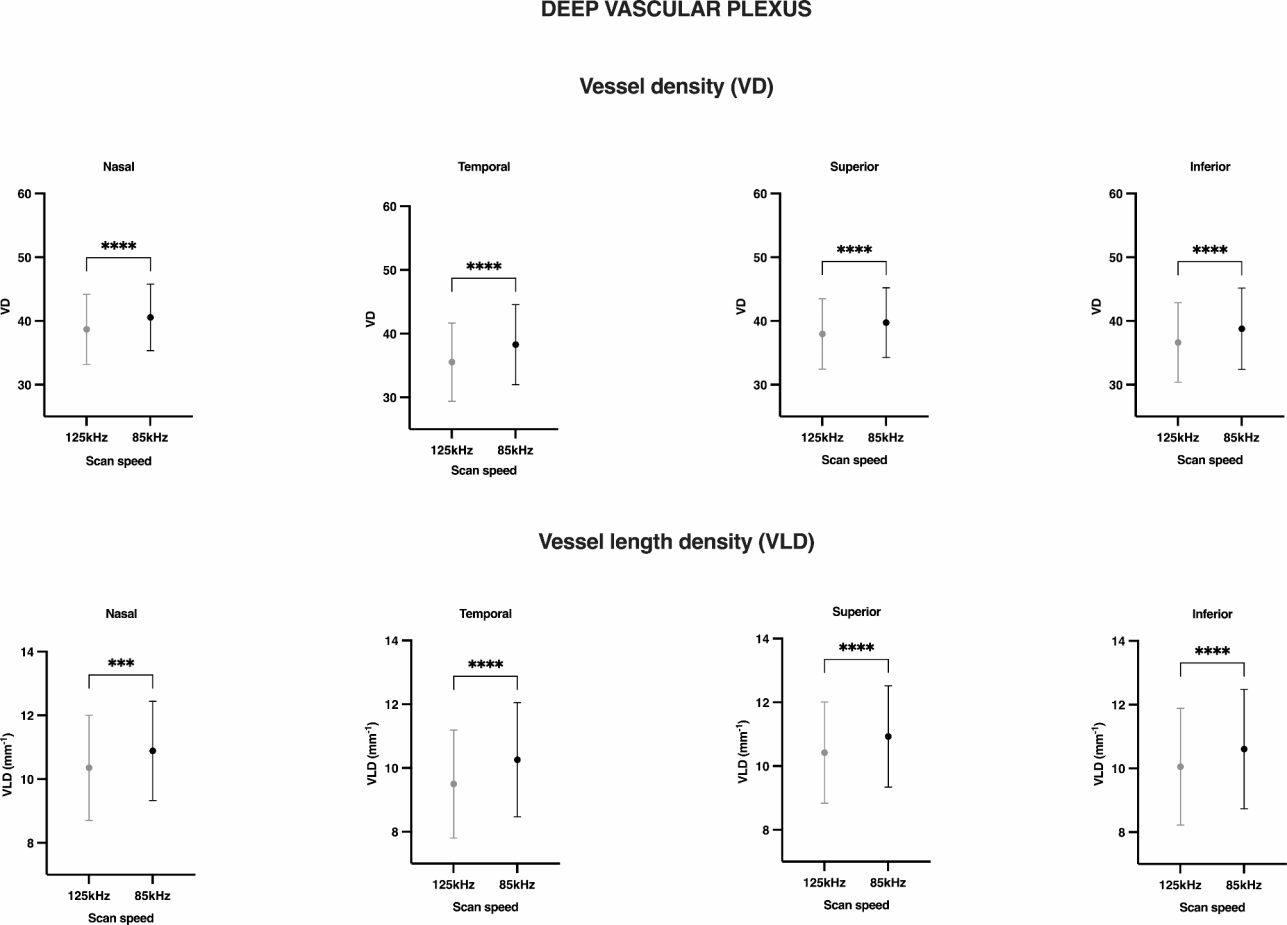
High acquisition speed alters deep vascular plexus metrics. Mean and standard deviation bars showing vessel density (VD, %) and vessel length density (VLD, mm^-1^) values of the deep vascular plexus in images taken at 125 kHz and 85 kHz scan speed, in different sectors of the retina. *P-values < 0.05, obtained by Wilcoxon matched-pairs signed rank test and corrected for multiple comparisons.

Both binarized and skeletonized vessel density showed significantly lower values in those images taken at 125 kHz scan speed in all quadrants around the fovea in either the superficial (Figs. 1 and 2) or deep vascular plexus (Fig. 3).

The VD reduction percentages were similar in both superficial and deep vascular plexus, with values ranging from − 4.03 to -5.8% in the SVP and − 3.35 to -6.58% in the DVP, depending on the retinal sector analyzed (Figs. 1 and 3). In the skeletonized images, the percentage change in VLD ranged from − 4.96 to -6.07% in the SVP and from − 3.60 to -6.66 in the DVP. In all cases, we observed a greater reduction in the temporal area (Table 1), but the differences between sectors were non-significant (SVP: VD *p* = 0.279, VLD *p* = 0.562; DVP: VD *p* = 0.246, VLD *p* = 0.581).

**Table 1:** Values of vessel density (VD) and vessel length density (VLD) in superficial and deep vascular plexuses of OCTA images taken at 85,000 and 125,000 A-scans/second (85 and 125 kHz).

	VD (%)				VLD (mm^− 1^)				
	85 kHz	125 kHz	% Reduction	*P*-value	85 KhZ		125 kHz	% Reduction	*P*-value
**Superficial vascular plexus**									
Nasal	46.65 ± 5.1	44.19 ± 5.31	-4.53 ± 6.23	< 0.0001*	11.47 ± 1.69		10.79 ± 1.69	-5.12 ± 7.99	< 0.0001*
Superior	46.42 ± 4.55	44.49 ± 4.48	-4.03 ± 5.37	< 0.0001*	10.92 ± 1.48		10.35 ± 1.53	-4.96 ± 7.5	< 0.0001*
Temporal	42.71 ± 5.57	40.03 ± 5.11	-5.8 ± 5.58	< 0.0001*	10.48 ± 1.72		9.8 ± 1.53	-6.07 ± 7.98	< 0.0001*
Inferior	46.34 ± 4.79	44.12 ± 5.07	-4.67 ± 6.93	< 0.0001*	10.89 ± 1.59		10.31 ± 1.61	5.18 ± 8.81	< 0.0001*
**Deep vascular plexus**									
Nasal	40.56 ± 5.22	38.69 ± 5.5	-3.35 ± 9,42	< 0.0001*	10.89 ± 1.55		10.36 ± 1.65	-3.69 ± 10.23	0.0002*
Superior	39.74 ± 5.45	37.96 ± 5.51	-4.07 ± 8.1	< 0.0001*	10.93 ± 1.59		10.42 ± 1.59	-4.36 ± 8.49	< 0.0001*
Temporal	38.27 ± 6.29	35.54 ± 6.14	-6.58 ± 7.91	< 0.0001*	10.26 ± 1.79		9.5 ± 1.7	-6.66 ± 8.82	< 0.0001*
Inferior	38.78 ± 6.4	36.61 ± 6.24	-4.88 ± 10	< 0.0001*	10.61 ± 1.87		10.05 ± 1.83	-4.49 ± 10.43	< 0.0001*

Mean values and percentage reduction using data from *en face* images taken at 85 kHz as baseline are shown. P-values obtained by Wilcoxon matched-pairs signed rank test. *False discovery rate < 0.05.

### Significance of contrast enhancement

In the images taken at 125 kHz, VD and VLD values increased following contrast enhancement through the utilization of both settings (range 5-149 and 10–144 pixels) in comparison to the image with unmodified contrast within the SVP (% change in VD: +0.78 ± 0.33 and + 1.11 ± 0.55; % change in VLD + 0.47 ± 0.43 and + 0.7 ± 0.53, *p* < 0.0001). Conversely, the values were observed to be lower in the DVP (% change in VD: -0.09 ± 0.6 and − 1.8 ± 1.0, *p* < 0.0001; % change in VLD − 0.04 ± 0.6, *p* = 0.005 and − 1.2 ± 0.98, *p* < 0.0001, respectively).

## Discussion

We found that an increase in acquisition speed resulted in significantly lower vessel density and vessel length density values in all areas of both superficial and deep vascular plexus. By enhancing image contrast these differences can only be partially evened out in the SVP, but not in the DVP.

To obtain the best possible OCTA images, both sensitivity to motion and image artifacts need to be considered. Longer interscan times will result in better sensitivity to slow flow and OCT signal intensity, but will introduce more artifacts due to eye movement.^12,13^ The interscan time depends on the A-scan rate, the sampling density, and the length of the B-scan.^14^ While keeping the other parameters constant, we changed the interscan time by increasing the A-scan rate (from 85 kHz to 125 kHz) using the commercially available SHIFT technology of the SPECTRALIS device. As a result, the sensitivity for detecting flow is also modified.

We found that this change in sensitivity results in a reduction of vascular metrics with faster A-scan rates in both the superficial and deep vascular plexus of a magnitude similar to the standard deviation of the respective measurements irrespective of scan location or scan direction. Enhancing image contrast prior to binarization of images captured at a higher scan speed partially evens out these discrepancies in the SVP, but not in the DVP. To the best of our knowledge, this is the first report on how scanning speed affects retinal vascular parameters in OCTA.

Despite us assessing only VD and VLD, we hypothesize that other metrics used to quantify the amount of vasculature in an area may be similarly affected, as most of them are variations or combinations of these two.^7^

As was to be expected, increasing the A-scan rate from 85 kHz to 125 kHz also reduced the acquisition time by 22%. In keeping with this, Dolz-Marco et al. reported a 28.5% reduction in acquisition time with 125 kHz and added that 92.5% of these OCTA images were rated as equal or better than the 85 kHz group in a qualitative assessment.^6^ Thus, faster scan speeds may be useful when acquiring images of a larger field of view or in patients with poorer cooperation, improving the performance of OCTA in the clinical setting. In addition, the shorter time intervals between B-scans may be more sensitive to faster flow (i.e., the choriocapillaris), and therefore its quantification may improve with faster A-scan rates.^15,16^

Our findings may be clinically relevant because VD values have been described as prognostic factors in many diseases such as intermediate uveitis, diabetic retinopathy or Parkinson’s disease.^17–19^ Although the VD quantification varies between manufacturers and image post-processing methods,^20,21^ the magnitude of the observed discrepancies is very comparable to that observed in many pathological changes, which merits careful attention when planning OCTA studies employing quantification of vascular parameters.

Strengths of our study include the prospective design and the homogeneous sample of patients with good retinal health and low spherical and astigmatic errors, which have been reported to affect image quality and thus vascular measurements.^11,22^ Furthermore, we took into account the impact of image contrast alterations as a potential source of bias.^23^ Our findings indicate that modifying this parameter may not be as crucial as optimizing acquisition speed.

Limitations of this study include the small sample size and the limited number of parameters investigated. It is unclear whether other vascularization metrics such as FAZ area or fractal dimension show similar sensitivity to scan speed. Furthermore, we analyzed only the superficial and deep vascular plexus and cannot comments on how different scan speeds may affect choroidal vascular measurements.

In conclusion, different OCTA scan speeds will lead to different quantitative measures of the SVP and DVP. Thus, scan speed should be reported when comparing quantitative parameters and kept the same within study samples and across time points. We recommend 85 kHz for evaluation of quantitative parameters in the research setting, especially for scans limited to the macular area, while faster scan speeds may have a place in a more clinical setting.

## Data Availability

The datasets used and/or analysed during the current study available from the corresponding author on reasonable request.

## References

[1] Choi, W. J. Imaging motion: A comprehensive review of optical coherence tomography angiography. *Adv. Exp. Med. Biol.* **1310**, 343–365 (2021). 33834441 10.1007/978-981-33-6064-8_12

[2] Onishi, A. C. & Fawzi, A. A. An overview of optical coherence tomography angiography and the posterior pole. *Ther. Adv. Ophthalmol.* **11**, 2515841419840249 (2019). 30984909 10.1177/2515841419840249 PMC6448101

[3] Tan, B. *et al.* Approaches to quantify optical coherence tomography angiography metrics. *Ann. Transl. Med.* **8**, 1205–1205 (2020). 33241054 10.21037/atm-20-3246 PMC7576021

[4] Coscas, G., Lupidi, M. & Coscas, F. Heidelberg Spectralis optical coherence tomography angiography: technical aspects. *Dev. Ophthalmol.* **56**, 1–5 (2016). 27022921 10.1159/000442768

[5] Spaide, R. F. *et al.* Optical coherence tomography angiography. *Prog. Retin. Eye Res.* **May**, 1–55 (2018). 10.1016/j.preteyeres.2017.11.003 PMC6404988 29229445

[6] Dolz-Marco, R., Muñoz-Solano, J., Dechent, J. F. & Gallego-Pinazo, R. The effect of increasing acquisition speed on optical coherence tomography angiography images: A qualitative and quantitative analysis. *Retina* **43**, 1653–1661 (2023). 37721724 10.1097/IAE.0000000000003867

[7] Kalra, G. *et al.* Optical coherence tomography (OCT) angiolytics: A review of OCT angiography quantitative biomarkers. *Surv. Ophthalmol.* **67**, 1118–1134 (2022). 34748794 10.1016/j.survophthal.2021.11.002

[8] Corradetti, G. *et al.* Choriocapillaris and retinal vascular alterations in presymptomatic Alzheimer’s disease. *Invest. Ophthalmol. Vis. Sci.* **65**, 47. 10.1167/iovs.65.1.47 (2024). 38294804 10.1167/iovs.65.1.47 PMC10839815

[9] Freedman, I. G. et al. The impact of image processing algorithms on optical coherence tomography angiography metrics and study conclusions in diabetic retinopathy. *Transl. Vis. Sci. Technol.* **11**, 7. 10.1167/tvst.11.9.7 (2022). 36107113 10.1167/tvst.11.9.7 PMC9483236

[10] Borrelli, E. *et al.* Optical coherence tomography angiography assessment of the diabetic macula: A comparison study among different algorithms. *Retina* **41**, 1799–1808 (2021). 33587426 10.1097/IAE.0000000000003145

[11] Vidal-Oliver, L., Gallego-Pinazo, R. & Dolz-Marco, R. Astigmatism influences quantitative and qualitative analysis in optical coherence tomography angiography imaging. *Transl. Vis. Sci. Technol.* **13**, 10. 10.1167/tvst.13.1.10 (2024). 38224331 10.1167/tvst.13.1.10 PMC10795549

[12] Cui, Y. *et al.* Imaging artifacts and segmentation errors with wide-field swept-source optical coherence tomography angiography in diabetic retinopathy. *Transl. Vis. Sci. Technol.* **8**10.1167/tvst.8.6.18 (2019). 10.1167/tvst.8.6.18 PMC6859832 31772829

[13] Kaizu, Y. *et al.* Longer interscan times in OCT angiography detect slower capillary flow in diabetic retinopathy. *Ophthalmol. Sci.* **2**, 100181 (2022). 36245749 10.1016/j.xops.2022.100181 PMC9560536

[14] Spaide, R. F., Fujimoto, J. G., Waheed, N. K., Sadda, S. R. & Staurenghi, G. Optical coherence tomography angiography. *Prog. Retin. Eye Res.* **64**, 1–55 (2018). 29229445 10.1016/j.preteyeres.2017.11.003 PMC6404988

[15] Migacz, J. V. *et al.* Megahertz-rate optical coherence tomography angiography improves the contrast of the choriocapillaris and choroid in human retinal imaging. *Biomed. Opt. Express* **10**, 50–65 (2019). 30775082 10.1364/BOE.10.000050 PMC6363198

[16] Choi, W. *et al.* Ultrahigh-Speed, Swept-Source Optical Coherence Tomography Angiography in Nonexudative Age-Related Macular Degeneration with Geographic Atrophy. *Ophthalmology* **122**, 2532–2544 (2015). 26481819 10.1016/j.ophtha.2015.08.029 PMC4658257

[17] Wintergerst, M. W. M. *et al.* Vessel density on optical coherence tomography angiography is prognostic for future disease course in intermediate uveitis. *Sci. Rep.* **14**, 2933 (2024). 38317017 10.1038/s41598-023-49926-0 PMC10844199

[18] Simonett, J. M. *et al.* Early microvascular retinal changes in optical coherence tomography angiography in patients with type 1 diabetes mellitus. *Acta Ophthalmol.* **95**, e751–e755 (2017). 28211261 10.1111/aos.13404

[19] Salehi, M. A. *et al.* Optical coherence tomography angiography measurements in Parkinson’s disease: A systematic review and meta-analysis. *Eye (Lond).* **37**, 3145–3156 (2023). 36941403 10.1038/s41433-023-02483-2 PMC10564940

[20] Arrigo, A. *et al.* The impact of different thresholds on optical coherence tomography angiography images binarization and quantitative metrics. *Sci. Rep.* **11**, 14758 (2021). 34285328 10.1038/s41598-021-94333-y PMC8292484

[21] Lu, Y. *et al.* A quantitative comparison of four optical coherence tomography angiography devices in healthy eyes. *Graefe’s Arch. Clin. Exp. Ophthalmol.* **259**, 1493–1501 (2021). 32975683 10.1007/s00417-020-04945-9

[22] Sampson, D. M. *et al.* Axial length variation impacts on superficial retinal vessel density and foveal avascular zone area measurements using optical coherence tomography angiography. *Investig. Ophthalmol. Vis. Sci.* **58**, 3065–3072. 10.1167/iovs.17-21551 (2017). 28622398 10.1167/iovs.17-21551

[23] Mehta, N. *et al.* Impact of binarization thresholding and brightness/contrast adjustment methodology on optical coherence tomography angiography image quantification. *Am. J. Ophthalmol.* **205**, 54–65 (2019). 30885708 10.1016/j.ajo.2019.03.008

